# Cyberinfrastructure for machine learning applications in agriculture: experiences, analysis, and vision

**DOI:** 10.3389/frai.2024.1496066

**Published:** 2025-01-23

**Authors:** Lucas Waltz, Sushma Katari, Chaeun Hong, Adit Anup, Julian Colbert, Anirudh Potlapally, Taylor Dill, Canaan Porter, John Engle, Christopher Stewart, Hari Subramoni, Scott Shearer, Raghu Machiraju, Osler Ortez, Laura Lindsey, Arnab Nandi, Sami Khanal

**Affiliations:** ^1^Department of Food, Agricultural, and Biological Engineering, The Ohio State University, Columbus, OH, United States; ^2^Department of Computer Science and Engineering, The Ohio State University, Columbus, OH, United States; ^3^Department of Horticulture and Crop Science, The Ohio State University, Columbus, OH, United States

**Keywords:** precision agriculture, multimodal data, machine learning, Unmanned Aerial Systems, crop phenotyping, cyberinfrastructure

## Abstract

**Introduction:**

Advancements in machine learning (ML) algorithms that make predictions from data without being explicitly programmed and the increased computational speeds of graphics processing units (GPUs) over the last decade have led to remarkable progress in the capabilities of ML. In many fields, including agriculture, this progress has outpaced the availability of sufficiently diverse and high-quality datasets, which now serve as a limiting factor. While many agricultural use cases appear feasible with current compute resources and ML algorithms, the lack of reusable hardware and software components, referred to as cyberinfrastructure (CI), for collecting, transmitting, cleaning, labeling, and training datasets is a major hindrance toward developing solutions to address agricultural use cases. This study focuses on addressing these challenges by exploring the collection, processing, and training of ML models using a multimodal dataset and providing a vision for agriculture-focused CI to accelerate innovation in the field.

**Methods:**

Data were collected during the 2023 growing season from three agricultural research locations across Ohio. The dataset includes 1 terabyte (TB) of multimodal data, comprising Unmanned Aerial System (UAS) imagery (RGB and multispectral), as well as soil and weather sensor data. The two primary crops studied were corn and soybean, which are the state's most widely cultivated crops. The data collected and processed from this study were used to train ML models to make predictions of crop growth stage, soil moisture, and final yield.

**Results:**

The exercise of processing this dataset resulted in four CI components that can be used to provide higher accuracy predictions in the agricultural domain. These components included (1) a UAS imagery pipeline that reduced processing time and improved image quality over standard methods, (2) a tabular data pipeline that aggregated data from multiple sources and temporal resolutions and aligned it with a common temporal resolution, (3) an approach to adapting the model architecture for a vision transformer (ViT) that incorporates agricultural domain expertise, and (4) a data visualization prototype that was used to identify outliers and improve trust in the data.

**Discussion:**

Further work will be aimed at maturing the CI components and implementing them on high performance computing (HPC). There are open questions as to how CI components like these can best be leveraged to serve the needs of the agricultural community to accelerate the development of ML applications in agriculture.

## 1 Introduction

In recent years, there has been a surge in interest across various domains in leveraging machine learning (ML) techniques to tackle complex, long-standing challenges. While technically a subfield of artificial intelligence (AI), the two terms, AI and ML, are often used interchangeably. ML is a branch of AI that focuses on developing algorithms and models that enable computers to learn from and make predictions or decisions based on data, without being explicitly programmed for specific tasks. AI refers to the broader field of creating systems or machines capable of performing tasks that typically require human intelligence and can include both rule-based systems that are explicitly programmed as well as learning-based systems like ML. In this article, we will specifically use the term ML for clarity and consistency. Since 2012, ML techniques have achieved remarkable milestones across multiple domains, such as AlexNet's victory in the ImageNet competition (Krizhevsky et al., 2012) and the introduction of the transformer architecture in 2017 (Vaswani et al., 2017). These milestones have propelled ML into unprecedented popularity.

This growing recognition of ML's potential has led experts in various domains to explore its applicability to their most daunting challenges. However, while the latest ML approaches are powerful, they perform best with extensive, high-quality datasets which are often expensive and labor-intensive to collect (Whang et al., 2023). Existing public datasets in agriculture, though useful, are often insufficient to harness the latest advances in model complexity and compute resources. Further, the process of collecting and processing agricultural data for ML faces numerous challenges, including sensor failures, data pipelines, and data privacy concerns.

The notion that data harnessed from agriculture, coupled with the latest advancements in ML, can significantly enhance both the profitability and sustainability of farming practices is not novel. Indeed, the agricultural industry's dominant players in seed, chemicals, fertilizer, and equipment along with tech companies and startups have invested heavily in farm management information systems (FMIS) to serve farmers. While many of these systems are focused on providing accurate records of past events, falling into the realm of descriptive analytics, there are increasing efforts to include predictive and prescriptive analytics into these software platforms using ML.

For example, Microsoft's Farmbeats project (Kapetanovic et al., 2017), launched in 2014, focuses on data-driven farming by integrating various data sources, like field sensors and UAS, to provide insightful analytics through computer vision and machine learning algorithms. It establishes an end-to-end Internet of Things (IoT) infrastructure for efficient data collection and utilizes TV white spaces for transmitting data to computing centers, thus enabling advanced data analytics, and, in turn, empowering farmers to enhance productivity and sustainability (Chandra et al., 2022). Another example is Mineral, originating from Google/Alphabet's X facility, which claims to have surveyed 10% of the world's farmland and developed 80 machine-learning models to boost production and mitigate agriculture's impact on the environment (Burwood-Taylor, 2023).

The creation of large-scale, high-quality multimodal datasets, carefully curated and made ready for ML applications, can significantly advance predictive and prescriptive analytics in agriculture. These datasets encompass spatial, spectral, and temporal dimensions. Spatial intensity refers to ground sampling distance (GSD) measured in centimeters or meters per pixel. Spectral resolution refers to the number of wavelength intervals, while temporal denotes the frequency of data collection. Gadiraju et al. (2020) demonstrated a 60% reduction in prediction error by using a multimodal deep-learning approach that leveraged spatial, spectral, and temporal data characteristics to identify crop types. This involved integrating a Convolutional Neural Network (CNN), often used for analyzing images, with spatially intensive data and a Long Short-Term Memory network (LSTM), often used to analyze text corpora, with highly temporal data. Presently, there is a growing research focus on data-driven agriculture systems that involve deploying a diverse array of IoT sensors for vast data generation and Big Data Analytics on these datasets (BDA) (Ur Rehman et al., 2019). This trend holds promise for enabling farmers to make more profitable and environmentally sustainable farming decisions. Furthermore, edge-cloud architectures (Taheri et al., 2023) can enhance real-time decision-making by hastening data processing.

In addition to the importance of data quantity, it is crucial to consider data quality prior to processing and incorporating data into model pipelines. The utilization of data quality indicators, such as data source, collection time, and environmental conditions, can serve to flag datasets with undesirable traits (Wang et al., 1993). These considerations underpin the critical role of data quality in agriculture's data-intensive domains.

The objective of this manuscript is to outline a vision for software and hardware infrastructure, or cyberinfrastructure (CI) that is oriented toward serving agricultural use cases.To illustrate the envisioned CI vision, we focus on three concrete and illustrative use cases: predicting soil moisture, crop growth stages, and yield. Examples of CI elements discussed in this paper include approaches to processing of Unmanned Aerial Systems (UAS) imagery that are optimized to reduce processing time, retain image quality, and increase spatial accuracy; use of application programming interfaces (API) to aggregate structured weather and *in-situ* sensor data; Vision Transformer (ViT) models adapted for agricultural use cases; and an interactive data visualization prototype to view geospatial data at various stages of the ML pipeline.

This proposed CI aims to facilitate the collection and processing of agricultural data at scale by providing a framework for reusable CI elements like those shared above that can run on a spectrum of hardware architectures from high performance computing (HPC) to edge processing.

While it is important to share these concrete examples, a more expansive vision is that a vibrant community sharing and exchanging CI components like these can create leverage that lowers the efforts to building the requisite datasets needed and subsequent creation of ML applications in agriculture, accelerating the training and inference of ML models that are ultimately used for the benefit of farmers and other stakeholders in agriculture.

## 2 Vision

Many agricultural use cases now appear to be within the capabilities of current compute resources and ML models. However, the lack of CI dedicated to the collection, transmission, cleaning, exploration, labeling, and training of the datasets (hereafter referred to as data pipelines), along with the challenges of deploying these solutions onto edge and intelligent sensing devices for inference are a major hindrance toward the development of solutions to address these use cases. Given the ongoing advancements in the ML community at large and the focused efforts within both agricultural industry and academia, we advocate for a vision to build publicly available agricultural datasets and the development of associated open source ML-centric CI. This CI would support the tools and resources necessary for agriculture-focused data pipelines. A vibrant open source community focused on CI and datasets for ML applications in agriculture (AgCI) has many positive benefits including:

1. Amplifies the efforts of agricultural researchers by reducing the time needed for building and debugging data pipelines, ultimately increasing the quality and quantity of their outputs and their extension efforts to farmers.
2. Connects computer science researchers with meaningful prevailing problems in the agricultural domain.
3. Lowers the capital requirements for startups to get to product market fit for ML based products and services in agriculture by leveraging open source software and datasets.
4. Enables positive economics for ML-based products for more of the long-tail of agricultural commodities beyond the dominant crops of corn, soybean, and wheat.

There are several companies that provide CI and other tooling to support ML initiatives in general. This includes the big three cloud providers (AWS, Azure, GCP) as well as companies such as HuggingFace, Kaggle, and Scale AI. However, the needs of agriculture are unique and can benefit from CI that is focused on salient agricultural use cases. There are several reasons for this assertion:

1. There are very few publicly available datasets of sufficient size and quality focused on agricultural use cases. Two examples are PlantVillage (J and Gopal, 2019) and a corn nitrogen research dataset (Ransom et al., 2021). PlantVillage contains 61,486 images comprising 39 different classes healthy and diseased plants. Its images are often taken in controlled settings with artificial backgrounds and may not be suitable for large-scale in-field inferencing. The lack of environmental context in these images limits their effectiveness in real-world agricultural settings, where factors such as background variability, lighting, and natural surroundings play a crucial role in model performance. The corn nitrogen research dataset contains 49 site-years from 2014–2016 across eight U.S. Midwest states. While these datasets are valuable for machine learning applications, they are limited in size and scope. However, there are many universities worldwide that collect volumes of agricultural data which if put in the right form, could be a tremendously valuable resource for ML model training.
2. The pipelines for collecting, transmitting, cleaning, and transforming agricultural data into formats ready for ML are labor-intensive and error-prone. Furthermore, agricultural researchers in many instances may not possess the data management and software development skills to effectively and efficiently perform these necessary tasks.
3. On-farm and small-plot research can be a rich source for training data. However, the collected ground truth labels may need to be modified to make them more suitable for ML model training. Furthermore, the approach for splitting the dataset into training, testing, and validation needs to consider the replications in the dataset. Failure to understand this could lead to data leakage where the test set performance is artificially improved because there are replicates from the same treatment in both training and test sets.
4. Commonly used ML models may need modifications to suit agricultural data. For instance, image-based ML models typically use a softmax layer as the final layer for classification. In agriculture, many outputs are measured on continuous scales, such as crop growth stages, disease severity, soil moisture, nutrient deficiency, and yield. Therefore, it might be valuable to evaluate both classification and regression-based approaches to determine which approach provides the best results for the specific use case.

**Figure 1:**
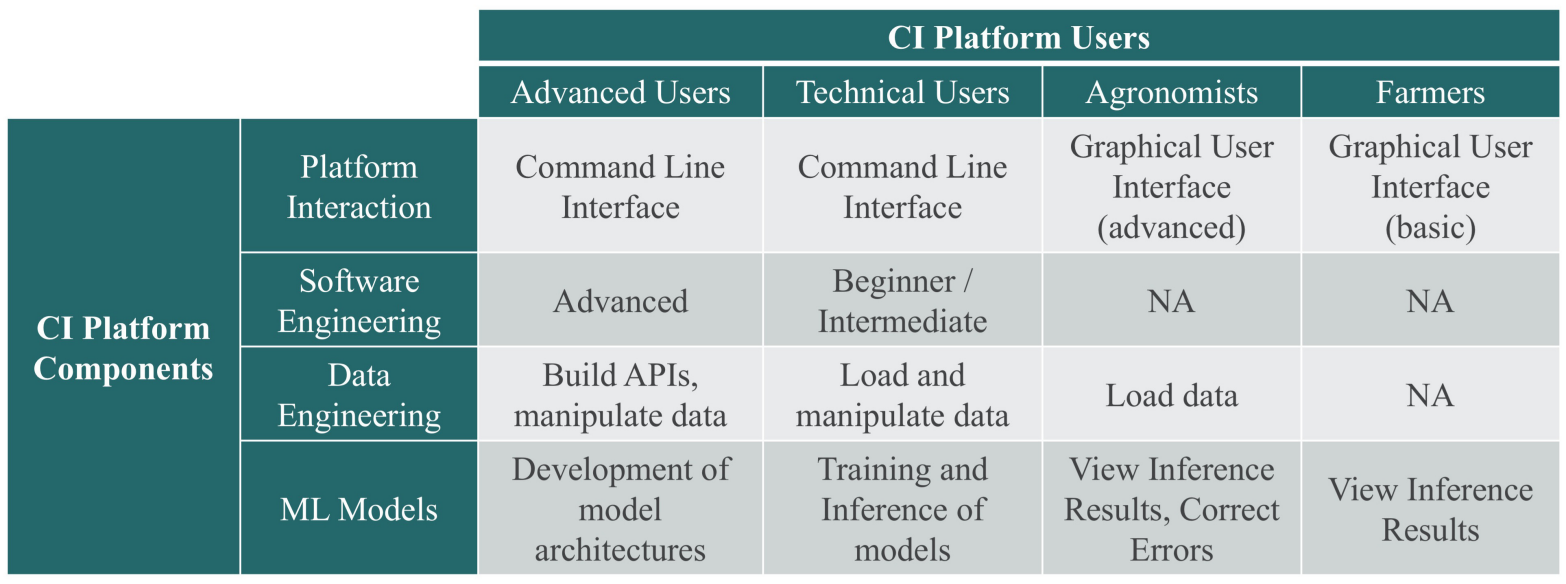
Mapping of cyberinfrastructure (CI) platform components to users.

For the reasons stated above, ML-amenable CI that leverages the capabilities in the ML community at large while adapting it for common use cases in agriculture has the potential to accelerate the benefits of ML in agriculture. With these benefits in mind, here are several core principles that guide our efforts to build AgCI that can enable more impactful ML applications in agriculture:

1. Data collection and CI efforts need to co-inform each other and should happen concurrently.
2. The speed for both training and inference are critical measures of value. Speed represents a holistic view that includes latency starting from the point at which data is collected in the field to the point where actionable insights are generated.
3. The CI should be capable of connecting to high-performance computing (HPC) to accelerate training and inference times.
4. The CI must incorporate the latest approaches and models from the broader ML community. Vision Transformers (ViT) (Han et al., 2023), semi- and weak-supervised labeling techniques (Sohn et al., 2020), and metadata formats (MLCommons, 2024) are examples. ViT techniques provide better adaptability, efficiency, and scalability, compared to traditional ML approaches that typically struggle with complex or spatially diverse agricultural imagery. Similarly, semi-and weak supervised labeling techniques allow models to learn from a few labeled images by leveraging unlabeled or weakly labeled data. In cases where labeled datasets are scarce and can be expensive to produce, this reduces the cost and time required in data annotation. Lastly, ML metadata formats such as Croissant seek to promote the discoverability and reusability of ML datasets.
5. The CI needs to be easy to use, trustworthy, and consider the range of technical proficiencies of potential users in agriculture. It also needs to include interfaces that provide transparency into the “black box” of ML and build confidence in its results. Figure 1 depicts a vision of the mapping of platform components to platform users.

**Figure 2:**
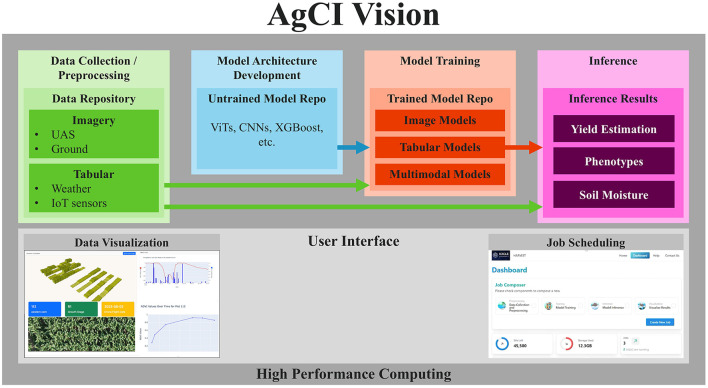
Description of important elements in AgCI.

The elements for AgCI are listed in Figure 2. It includes: (1) Data Collection/Preprocessing pipelines to turn raw data into ML-ready data structures. Imagery from unmanned aerial systems (UAS) and smartphones, along with tabular data from weather stations and IoT sensors, were utilized in the selected use case; (2) ML Model Architecture Development which includes repositories of untrained models, such as ViTs, CNNs, and XGBoost, that have been optimized for agricultural use cases; (3) Repositories of Trained Models; and (4) Inferences/recommendations generated from trained ML models. A User Interface is important for each step. Two important use cases for a User Interface are Data Visualization to provide human feedback that Data Collection/Preprocessing pipelines have correctly transformed the data and Job Scheduling to schedule and initiate jobs for different elements of the workflow. Lastly, our vision is that these elements need to be built on a high-performance computing (HPC) backbone to reduce the time needed for ML model training and inference on sizable datasets.

It is important that visions be grounded in reality and informed by continuous testing and feedback. Collins (2001) highlights this in *Good to Great*, emphasizing the importance of embracing the Stockdale Paradox by confronting the brutal facts while maintaining faith in the end goal. Similarly, Ries (2011) advocates in *The Lean Startup* for the importance of validated learning as a tool to make constant adjustments to a vision. While these books advocated approaches for companies, we think they also have valuable application for the subject matter of this paper. With that in mind, the following sections highlight our experiences in building data pipelines for three important use cases in agriculture, namely yield estimation, growth stage prediction, and soil moisture prediction, and serve as an important source of feedback in refining our vision for AgCI. While the paper focuses on learnings from three specific use cases, we believe our vision for AgCI can be useful for a much broader range of agricultural use cases that can benefit from ML approaches.

We note that the proposed vision in this manuscript is particularly relevant to those land-grant universities in the United States who were formed via the Morrill Land-Grant Acts of 1862 and 1890 “to teach such branches of learning as are related to agriculture." This mission was strengthened via the Hatch Act of 1887 that established funding for agricultural experiment stations and the Smith-Lever Act of 1914 that established the Cooperative Extension Service as a means of “diffusing among the people of the United States useful and practical information on subjects relating to agriculture." While the authors of these legislative acts could not possibly have imagined the advancements in agriculture that would have happened over the last 150 years, our vision of establishing AgCI strongly aligns with the foundation they laid of establishing land-grant universities with a mission to promote agricultural advancement for the benefit of society.

## 3 Materials and methods

### 3.1 Initial data types

**Figure 3:**
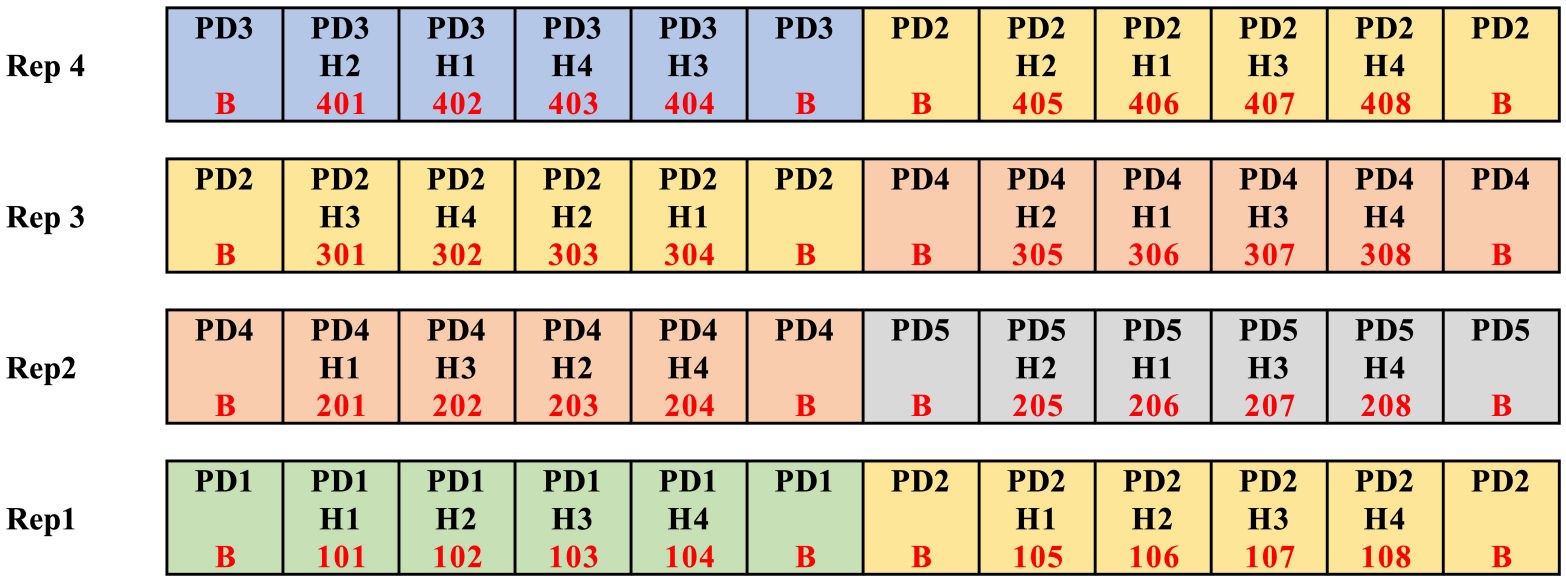
Portion of corn plot map for Western Agricultural Research Station.

**Figure 4:**
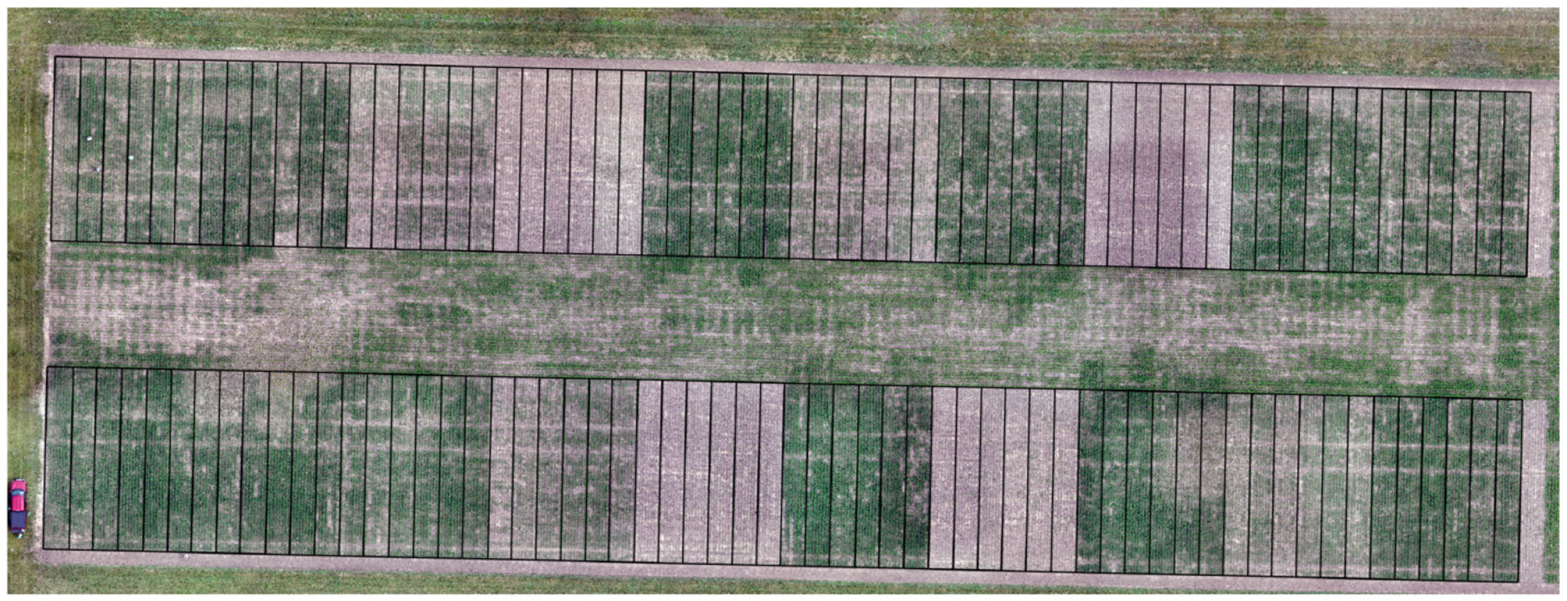
Plots from Northwest Agricultural Research Station overlaid on orthomosaic from UAS imagery.

The initial data sources that provide feedback to our AgCI efforts originate from three agricultural research stations geographically dispersed across Ohio and operated by The Ohio State University (OSU). They include Western Agricultural Research Station in Clark County, Northwest Agricultural Research Station in Wood County, and Wooster Campus in Wayne County. Each site included 80 plots for corn and 80 plots for soybean. The experiment was a split-plot randomized complete block design with four replications of each treatment. Main plot factor included five planting dates spaced approximately every 2 weeks from mid-April to mid-June. Figure 3 shows an excerpt of the plot map for the corn plots in Western Agricultural Research Station. Figure 4 shows plots from Northwest Agricultural Research Station overlaid on an orthomosaic processed from UAS imagery.

**Figure 5:**
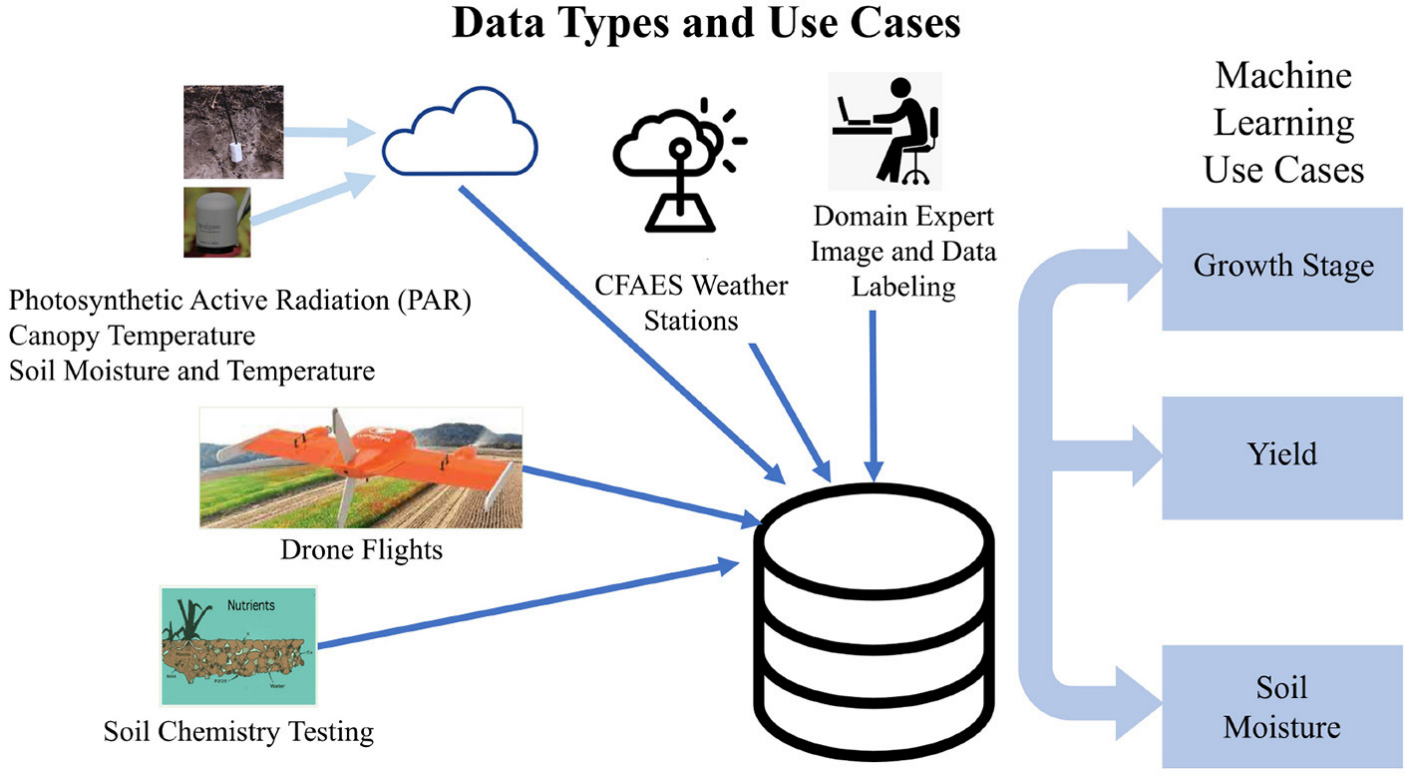
Summary of initial data types and use cases.

The subplot factor for corn consisted of four hybrids with four different relative maturities (100-, 107-, 111-, and 115-day), while the subplot factor for soybean involved four seeding rates (247,000; 345,800; 444,600; and 518,700 seeds per hectare, equivalent to 100,000; 140,000; 180,000; and 210,000 seeds per acre, respectively). Each replicate included a border plot on both ends of the block to reduce any edge-of-field effects on the measured plots. Furthermore, yield measurements were based on the center two rows (out of four) for corn and the center five rows (out of seven or eight) for soybean. The research plots were managed according to agronomic best management practices for soybean (Lindsey et al., 2017) and corn (Thomison et al., 2017) outside of the main plot and subplot factors. Figure 5 is a summary diagram that shows initial data types collected and initial use cases.

In total, × 1 terabyte (TB) of data were collected, with the vast majority of that being from UAS imagery. While a dataset size of 1 TB may not be considered extensive according to contemporary standards, it signifies a substantial investment in terms of time and labor in the agricultural domain. The individual data types are enumerated below.

#### 3.1.1 Unmanned Aerial Systems imagery

The aerial image collection was facilitated using a Wingtra One Unmanned Aerial System (UAS), equipped with both a 42MP RGB camera, the Sony RX1R II, and a Micasense Altum Multi-spectral camera featuring six spectral bands: Red, Green, Blue, Red-edge, Near Infrared, and Thermal Infrared. Flight missions for the Sony RX1R II were conducted at an altitude of 50 m above ground level (AGL), resulting in a ground sampling distance (GSD) of 0.008 m/pixel. The Micasense Altum was flown at 70 m AGL, resulting in a GSD of 0.047 m/pixel. Flight missions were executed at approximately weekly intervals throughout the entire growing season, beginning in May 2023 and culminating with the final flights in mid-October 2023 shortly before harvest. This strategy resulted in a total of between 13 and 16 flights per site for each camera. Each flight mission generated hundreds of images covering the corn and soybean plots at each research location.

#### 3.1.2 Structured soil and climate data

##### 3.1.2.1 *In-situ* soil and weather sensing data

An array of soil sensors was deployed at two depths, specifically at 30 and 60 cm, within the corn and soybean plots for both Planting Date 2 (26-27 April 2023) and Planting Date 4 (25–30 May 2023) at all three research locations. These soil sensors included Teros 12 (volumetric water content (VWC), soil temperature (ST), and electrical conductivity (EC)) and Teros 21 (matric potential (MP)) sensors at 30 cm depth and Teros 11 (VWC and ST) at 60 cm depth. Additionally, one Apogee SQ-521 photosynthetic active radiation (PAR) sensor and one Meter ATMOS 14 weather station were installed at each of these research sites. The ATMOS 14 weather stations collected temperature, relative humidity, vapor pressure, and barometric pressure in the crop canopy. The installation of these sensors occurred at all three sites in early June 2023 and collected data until shortly before harvest in mid-October 2023 at a 30-min temporal resolution.

The data collected by these sensors were aggregated by a total of six data loggers, with two loggers allocated at each research site. These loggers were connected to the Meter Group's Zentra Cloud, a data management and visualization platform. Data visualization was available through user-configurable dashboards on the website and data were also accessible via an application programming interface (API).

##### 3.1.2.2 Weather station data

At each of the research locations, an OSU managed weather station collects precipitation, wind speed, and air temperature at multiple heights, which is accessible at weather.cfaes.osu.edu. In addition, the website also provides calculated daily values such as heat units, commonly expressed as Growing Degree Days (GDD), using the following formula (McMaster and Wilhelm, 1997):


$$\text{GDD}=\left(\frac{T_{\text{max}}+T_{\text{min}}}{2}\right)-T_{\text{base}}$$

The base temperature for corn is typically set at 10°C. The accumulation of GDD over the growing season is widely used in predicting corn growth and development (sometimes referred to as heat units accumulation). The weather data is available year-round at both 5-min and daily temporal resolution for a number of OSU research locations including the three that were part of this study. This study used the daily temporal resolution data.

##### 3.1.2.3 Soil testing data

At the beginning of the growing season in May 2023, standard soil chemistry and soil texture tests were conducted for each location. Soil chemistry tests provide values of the concentrations of various nutrients, percentage of organic matter (OM), and cation exchange capacity (CEC). Soil texture tests measure the percentage of sand, silt, and clay. On an approximately weekly basis coinciding with the UAS flight missions, nine soil samples were taken from each plot at a depth of 15 cm corresponding to the locations of the *in-situ* soil and climate sensors. These samples were aggregated together for each plot and submitted to a soil testing laboratory to measure plant-available nitrogen content, consisting of nitrate and ammonium, as well as CO_2_ respiration reported in mg/kg as an indication of the rate of nitrogen mineralization of organic matter.

##### 3.1.2.4 Ground-truth data

Similarly, weekly site visits from May to October 2023 were conducted at all three research locations by personnel from the OSU's Department of Horticulture and Crop Science (HCS). These individuals possessed expertise in the classification of corn (Hanway, 1963) and soybean (Fehr et al., 1971) growth stages as well as proficiency in assessing disease incidence and quantifying disease severity. Furthermore, ears of corn and whole soybean plants with pods were collected at harvest for detailed measurements of the components of yield such as kernel rows, kernels per row and kernel weight in corn and seeds per pod, pods per plant, and seed weight in soybean. The data generated from these site visits will be the labels for several ML use cases derived from this data set.

### 3.2 Initial use cases

#### 3.2.1 Growth stage prediction

Growth stages are an objective way to track the progress of corn and soybean from emergence through to maturity. The availability of water and nutrients in each growth stage can be an important predictor of yield. Furthermore, certain treatments for plant disease can be more effective if applied during certain growth stages. While temperature-based calculations such as GDD accumulated since planting date can be used as a way to estimate growth stages, in some cases, the planting date may not be known. Additionally, drought stress or time of the year at which the crop is planted (Nielsen, 2022) can reduce the accuracy of GDD-based growth stage predictions. With the increasing prevalence of UAS-based imagery, this use case focuses on the use of Vision Transformers (ViT) to estimate growth stage from UAS images. We used both classification and regression-based approaches to predict 16 growth stages in corn from V1 to R5.

#### 3.2.2 Soil moisture

Soil moisture is an important attribute for both rainfed and irrigated crops. The flow of water via transpiration from the roots through to the stomata is a necessary requirement for photosynthesis and is also the transport mechanism for important nutrients such as nitrogen, phosphorus, and potassium. Furthermore, the water balance in the soil has a strong influence on the soil nitrogen budget and optimum nitrogen fertilizer rates. Understanding soil moisture is valuable, therefore, for informing nutrient management and estimating yield. This use case seeks to predict soil moisture for different soil textures based on weather and crop growth stages.

The approach used a Long Short-Term Memory model to predict the daily change in volumetric water content and thus predict a running account of total volumetric water content in the soil.

#### 3.2.3 Yield estimation

Yield estimation is valuable in-season for informing farmers' grain marketing decisions. If they have a better estimate of their yield, they can choose to lock-in pricing for a greater amount of their harvest. Furthermore, estimates of yield potential during the growing season can inform the profitability of field treatments such as nitrogen and fungicide applications. In this approach, we used a Long Short-Term Memory architecture to predict yield across 228 plots at the three research locations using a leave one out (LOO) cross-validation approach for the five planting dates (Waltz et al., 2024).

**Figure 6:**
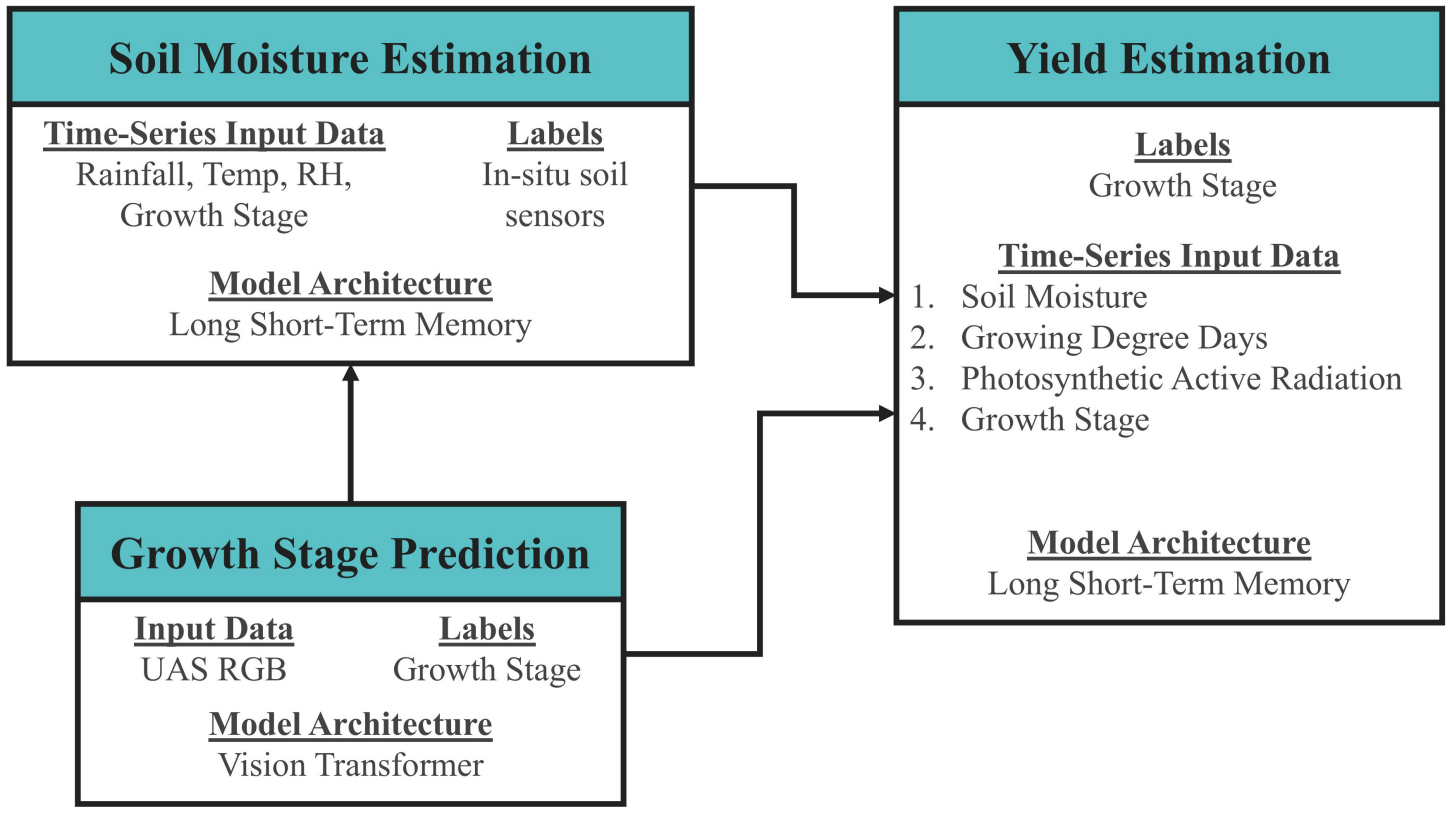
Interconnected machine learning models for yield estimation.

While the yield estimation model used the actual ground truth labels for growth stage and running averages of precipitation as inputs, Figure 6 shows how the three use cases could be combined in the future with interconnected ML models to estimate yield in a more integrated fashion that would be more amenable to being deployed at scale. The results of soil moisture and growth stage ML models can be combined with growing degree days and photosynthetic active radiation during the growing season to estimate yield.

## 4 Results

This section outlines the creation of CI components that are important for developing the use cases in Section 3.2. This includes an imagery and tabular data pipeline, a modified ViT model architecture better suited to agricultural data, and a data visualization prototype to improve trust and identify outliers in the dataset. While these CI components are important for three identified use cases, there are many other agricultural use cases that could potentially benefit from these CI components.

### 4.1 UAS imagery pipeline

Over the course of the 2023 growing season, a total of 85 flight missions were conducted. These missions included flights across three research locations utilizing two payload sensors, namely RGB and multi-spectral. Making use of commercially available products, our UAS-based data acquisition relied on the use of Secure Digital (SD) cards to store images captured during UAS missions and subsequently transferred to a hard drive, where proprietary software was utilized to geotag images from either the nearest Continuously Operating Reference Station (CORS) or an on-site Global Navigation Satellite System (GNSS) receiver. CORS generate correction signals, including Virtual Reference Station (VRS) signals, which are used to improve GNSS receiver position accuracy. This can be achieved through Real-Time Kinematic (RTK) or Post-Processed Kinematic (PPK). The Wingtra One utilized PPK to improve the spatial accuracy from meter to centimeter level accuracy.

Our initial approach used Pix4D, a commercial photogrammetry software provider, to generate orthomosaics from each flight. Specifically, Pix4Dengine, a set of programming modules, facilitated the automation of the orthomosaic creation through a Python script. Our data pipeline also involved the creation of plot boundaries in the form of polygon shapefiles (.shp) corresponding to the geographic coordinates of each plot. These shapefiles were used as a mask to create images for each plot from each flight.

**Figure 7:**
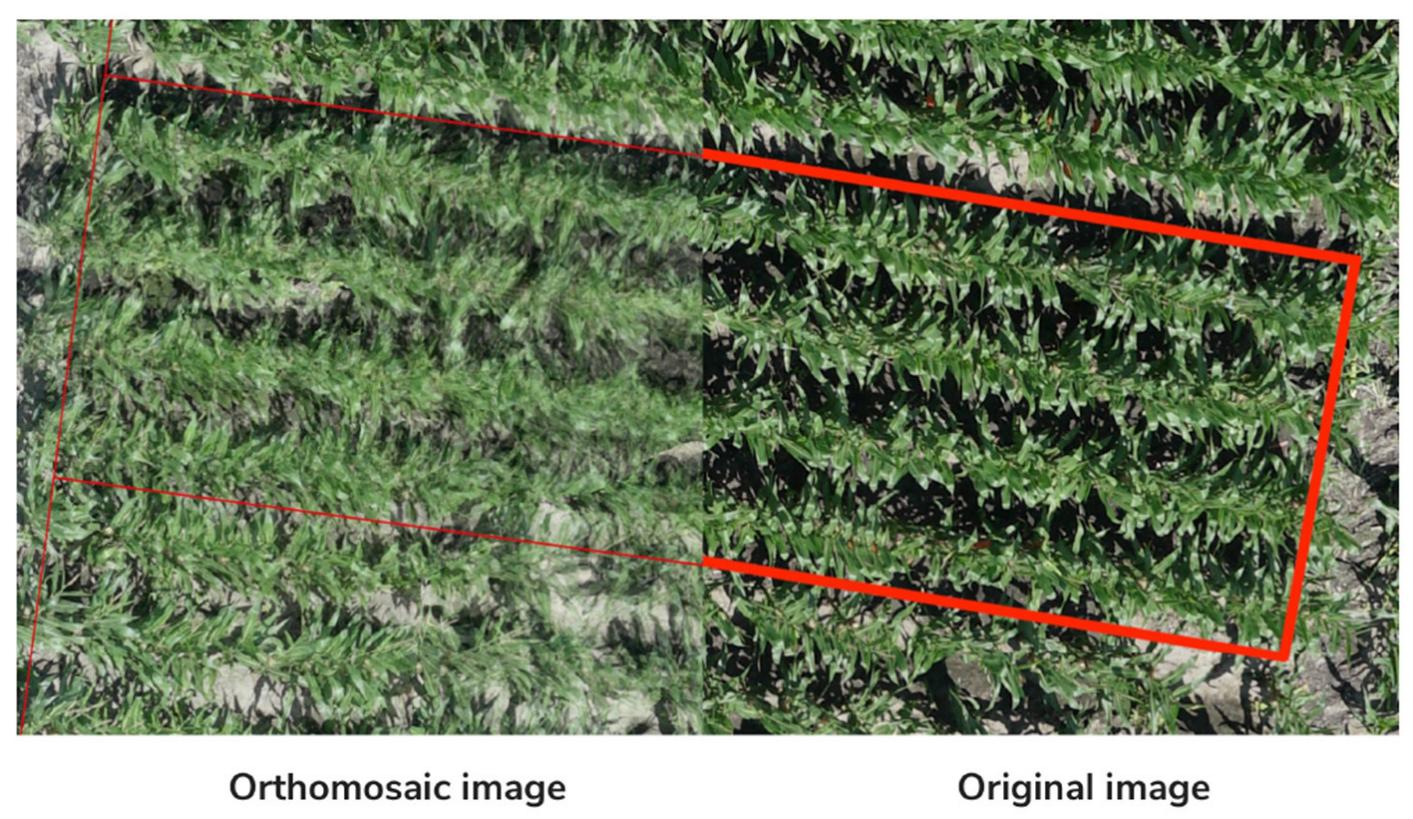
Illustration of degradation in image quality comparing orthomosaic to original image.

During this process, we experienced several challenges. First was that the orthomosaic creation was a lengthy process, generally taking 4–6 h to complete. This corresponded to × 25 min of processing time per hectare. Secondly, the stitching process caused degradation in the resulting orthomosaic's image quality, partially due to motion artifacts caused by the movement of corn and soybean plants in overlapping regions of successive images. Lastly, in our first attempt to create orthomosaics, we experienced roughly 10% of the orthomosaics were incomplete and did not cover the entire plot area. While we were able to get these orthomosaics to cover the entire area by adjusting the settings on Pix4D, this process increased processing time from 4–6 h to 1–2 days. Figure 7 illustrates the degradation in image quality that can occur while generating an orthomosaic.

Since the settings required to generate orthomosaics involved lengthy processing times and yet the resulting image quality was often significantly degraded from the underlying raw image, an alternative approach was developed with the primary goal of retaining the original image quality.

The new approach utilized a technique called direct georeferencing that utilizes the translational (latitude, longitude, and altitude) and rotational (roll, pitch, and yaw) orientation of the UAS that is associated with each geotagged image to georeference each image individually. While this approach improved image quality and reduced processing time, it came at the expense of reduced geospatial accuracy from a mean error of 0.003 m to 0.5 m. While this accuracy would be suitable for training data from on-farm research where treatment sizes are typically >12 m wide by 100 m long, it was not suitable for small-plot research with typical plot sizes of 3 m wide by 10 m long.

To address this reduced geospatial accuracy, we added a step of image registration where the direct georeferenced image was registered against the orthomosaic. This resulted in a georeferenced image that achieved a mean error of 0.06 m that was suitable for small-plot research.

**Figure 8:**
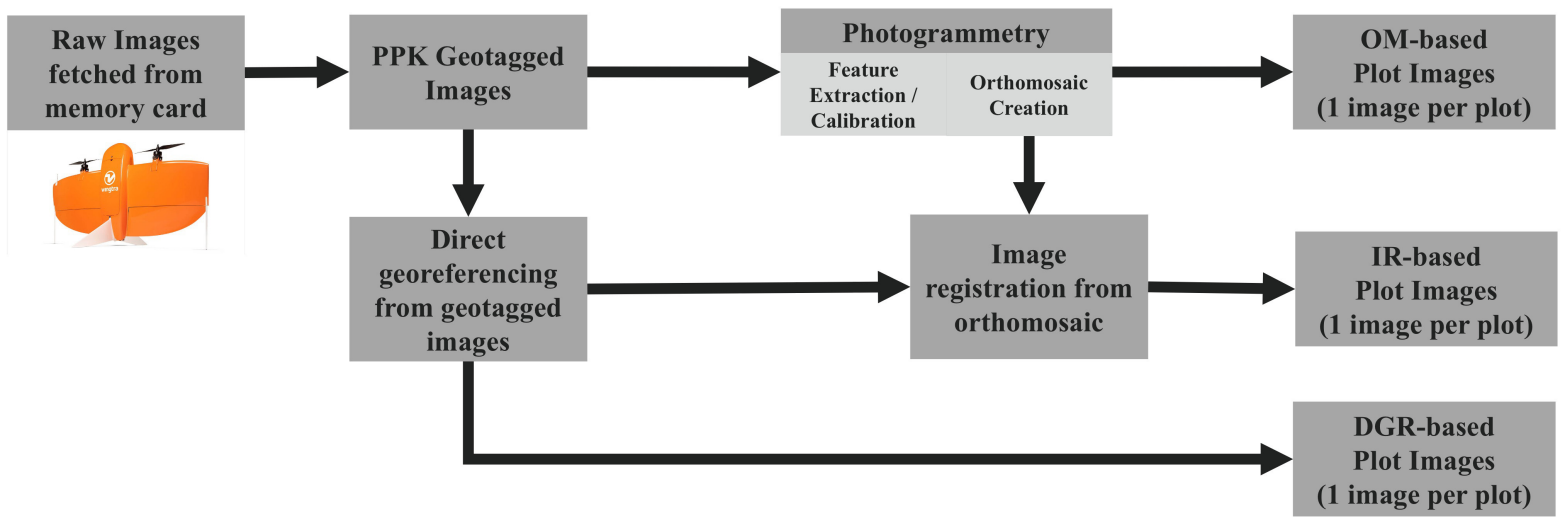
Illustration of portfolio of approaches to process UAS imagery into ML ready images including Orthomosaic (OM-based), Image Registration (IR-based), and Direct Georeferencing (DGR-based).

Figure 8 illustrates the three approaches that were evaluated to generate georeferenced plot images corresponding to agronomic treatments in small-plot research trials. Table 1 compares the three approaches with performance metrics of processing time, geospatial accuracy, and image quality.

**Table 1:** Comparison of UAS image processing performance using different pipelines.

**Metric**	**Orthomosaic pipeline (baseline)**	**Direct georeferencing pipeline**	**Image registration pipeline**
Application		On-farm research	Small-plot research
Processing time from raw images to plot tiles	~25*min*/*ha*	~3*min*/*ha*	~30*min*/*ha*
Geospatial accuracy (mean error)	0.003 m	0.5 m	0.06 m
Image degradation	Stitching artifacts	Minimal	Minimal

The new pipelines designed for automated creation of tiles for each plot represent improvements over the orthomosaic baseline approach. The Direct Georeferencing Pipeline results in an eight-fold improvement in processing time while simultaneously eliminating stitching artifacts that degrade image quality. While there is a decrease in geospatial accuracy, this approach is well-suited to on-farm research where a 0.5 m accuracy is acceptable.

For small-plot research where higher geospatial accuracy is needed, the Image Registration Pipeline is able to eliminate the stitching artifacts that degrade image quality while achieving similar geospatial accuracy and processing times from the Orthomosaic Pipeline. The improved image quality is expected to boost the accuracy of machine learning models, while faster processing time makes both training and inference more efficient.

In addition to the quality and processing time improvements, automated pipelines eliminate the human bottleneck that is often the largest contributor to the latency from the point of image collection to those images being available for ML training.

### 4.2 Soil and weather structured data pipeline

**Figure 9:**
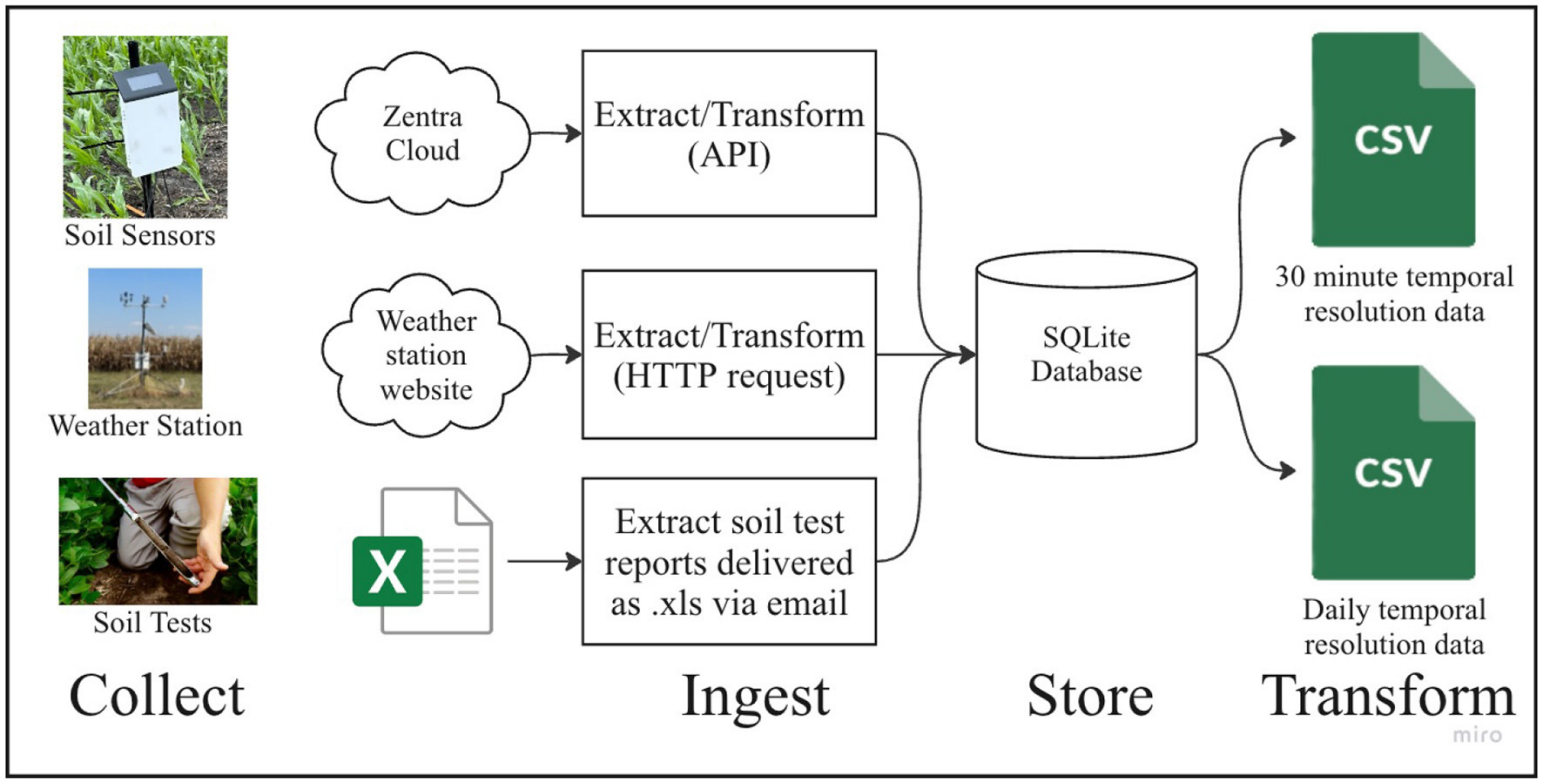
Soil and weather structured data pipeline.

The Soil and Weather Structured Data Pipeline aggregates structured data from three separate sources (soil sensors, weather stations, and soil lab tests) into a database as shown in Figure 9.

This pipeline harnessed data from *in-situ* field sensors, including soil volumetric water content, soil matric potential, photosynthetic active radiation, temperature, and relative humidity. These sensors were connected to Meter ZL6 loggers, which recorded data at 30-min intervals. Subsequently, the collected data was transmitted to the Meter Zentra Cloud through cellular connections. A Python script was employed to interface with the Zentra Cloud application programming interface (API) to retrieve the data and aggregate it into a local database.

Additionally, the pipeline incorporated data from OSU weather stations, which were located at each research site near the field plots. The data generated by these weather stations was accessible via a web interface, allowing for convenient querying and retrieval.

Soil lab testing results were received regularly as spreadsheets sent over email. The data in these spreadsheets were also incorporated into the database.

Our data cleaning process was heavily reliant on the use of Jupyter notebooks to manually handle CSV and Excel files, involving unique scripts for each type of data transformation required such as mapping growth stage descriptions to numeric values, converting irregular time-series data into a standardized daily format, and averaging hourly sensor readings to daily values.

While this enabled batch processing of data at the end of the growing season, the next step of maturity in the data pipeline is to implement continuous data processing. To accomplish this, we envision a data pipeline that would replace these manual Jupyter notebook operations with integrated, automated tasks enabling continuous data processing capabilities. This architecture would incorporate an Extract, Transform, and Load (ETL) scheme scheduled to operate continuously. As new data arrives, it would be extracted and then transformed by applying various cleaning techniques such as normalizing time-series data into a uniform temporal resolution and aligning disparate data formats into a unified format. Following transformation, the data would be loaded into a database which would act as the central repository from which the front-end user application can dynamically query the database and retrieve data on-demand.

### 4.3 Model architecture

**Figure 10:**
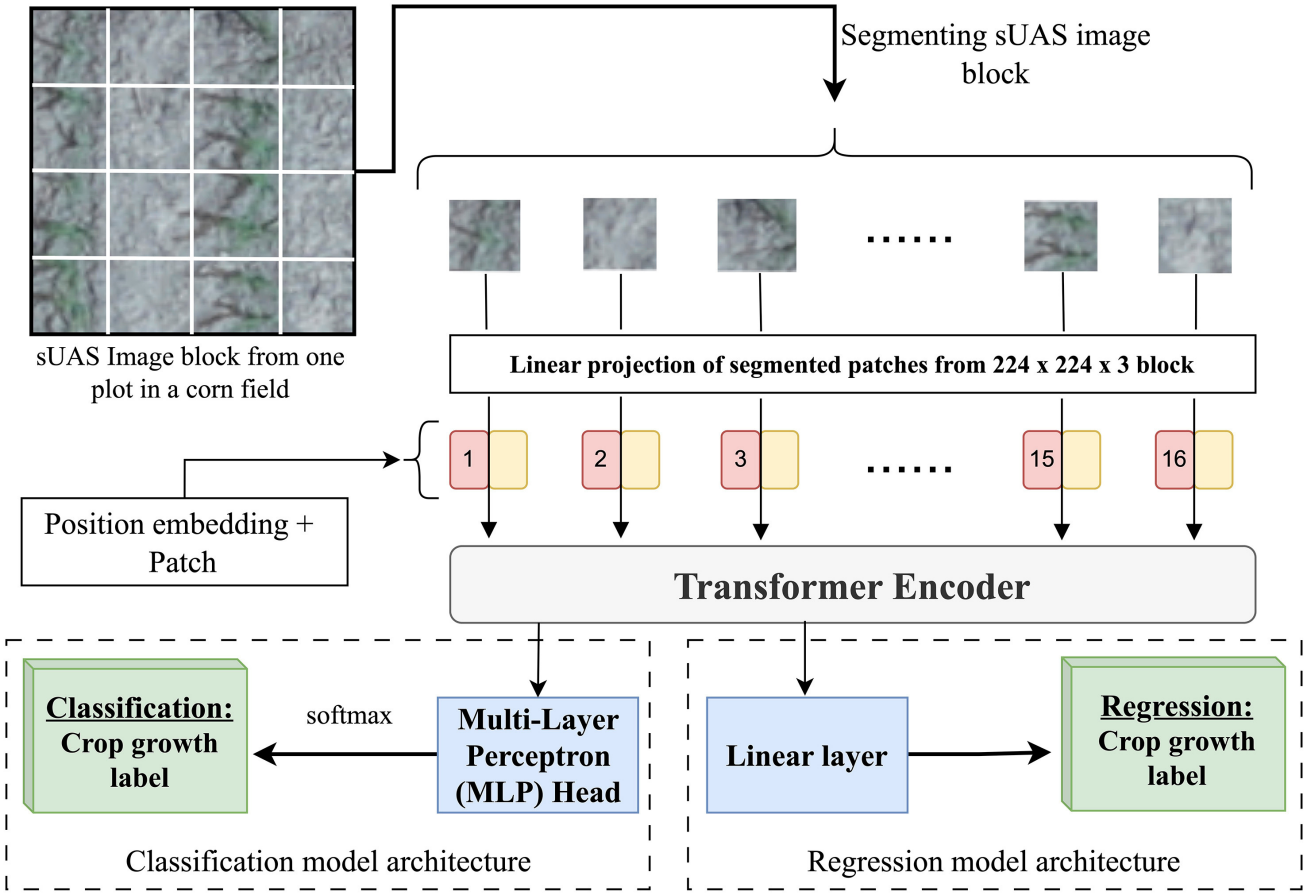
Illustrating steps in training the Vision Transformer (ViT) model using UAS images to identify crop growth stages as classification and regression tasks.

After acquiring and preprocessing various data types using data pipelines, the next step is ML model training. ML models such as Support Vector Machines (SVM), decision trees, regression networks, Convolutional Neural Networks (CNN), and Long Short Term Memory networks (LSTM) are popularly selected for various agricultural use cases (Khanal et al., 2020). In this study, the state-of-the-art Vision Transformer (ViT) model was used to identify corn growth stages using UAS RGB images. Studies (Han et al., 2023) have found ViT models to perform similar to or better than other types of neural network (NN) based models such as CNN and recurrent RNN. They better capture spatial relationships, such as the development of leaves and the presence of flowers in the images, which can be crucial to identify crop growth stages. To compare the classification and regression approaches for estimating crop development, the ViT architecture was modified to perform these tasks (Figure 10). During the training of the classification model, each crop growth stage is treated as an independent, discrete observation, whereas in a regression model, crop growth is considered a continuous observation.

After the model selection, the input data can either be segmented or resampled to match the model specifications and requirements. For the ViT model, UAS images from each of the plots were divided into blocks of 224 × 224 × 3 to meet the ViT input requirement and then passed to the position embedding layer of ViT architecture. The embedded data is then passed to the transformer encoder and then to either the Multi-Layer Perceptron (MLP) head (classification) or Linear layer (regression). The MLP head and Linear layer use categorical cross entropy and mean squared error (MSE) as the loss function, respectively.

Each of the 224 × 224 × 3 blocks were annotated with ground-observed crop growth labels from their respective plot. These alphanumeric labels represent specific stages in corn growth cycle, with vegetative and reproductive stages denoted with the prefix “V” and “R” respectively. These labels can be considered as independent, discrete labels (classification) or as a sequence of continuous labels (regression). The alphanumeric growth stage labels from V1 to R6 were converted to numeric values from 0 to 22. The regression predictions were rounded to the nearest integer.

As we evaluated the dataset, we discovered that there was significant class imbalance with growth stages V10 to VT having many fewer samples than the other classes. On further reflection, this related to the fact that the ground truth sampling frequency was approximately weekly. However, while the growth stages prior to V10 and subsequent to R1 transitioned approximately once every five to seven days, the growth stages from V10 to VT happen much more quickly at a rate of 1 to 3 days on average. The result of this was that our dataset contained many fewer ground truth observations per class from V10 to VT. Furthermore, when looked at from an agricultural perspective, the importance of distinguishing between individual growth stages from V10 to VT carries less practical value for our yield estimation use case. For these reasons, we evaluated model performance with the existing classes and with a consolidated approach that grouped the classes from V10 to VT into 2 groupings: V10–V12 and V13–VT. This provided benefits from a technical perspective by resulting in more balanced classes and from a pragmatic standpoint in that the finer grained detail does not provide additional downstream benefits. This highlights the importance of synthesizing domain expertise in agronomy with technical expertise in ML to arrive at better solutions.

**Figure 11:**
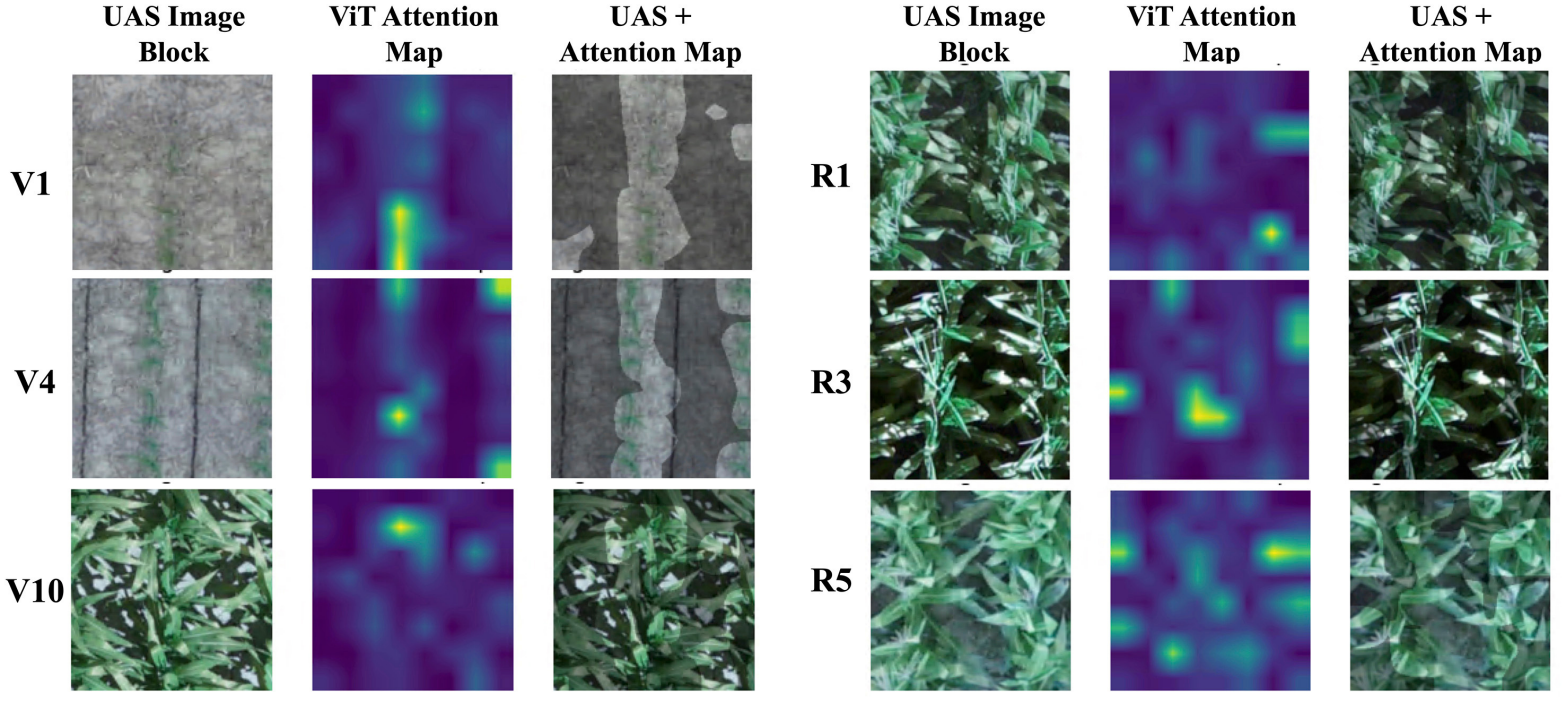
Examples of UAS image blocks (224 × 224), attention maps, and attention maps overlaid on UAS image blocks.

The images were randomly partitioned into 80% for training and 20% for testing. Figure 11 shows examples of UAS image blocks (224 × 224 × 3) of selected growth stages along with the associated attention maps, and attention maps overlaid on top of UAS image blocks. Attention maps highlight the areas of an image that the ML model learns from.

With two different approaches to growth stage labels (grouped and non-grouped) and two different approaches to the final layer in the ViT model architecture (classification and regression), we evaluated four combinations and measured the performance of each approach with respect to classification accuracy and mean squared error (MSE).

**Figure 12:**
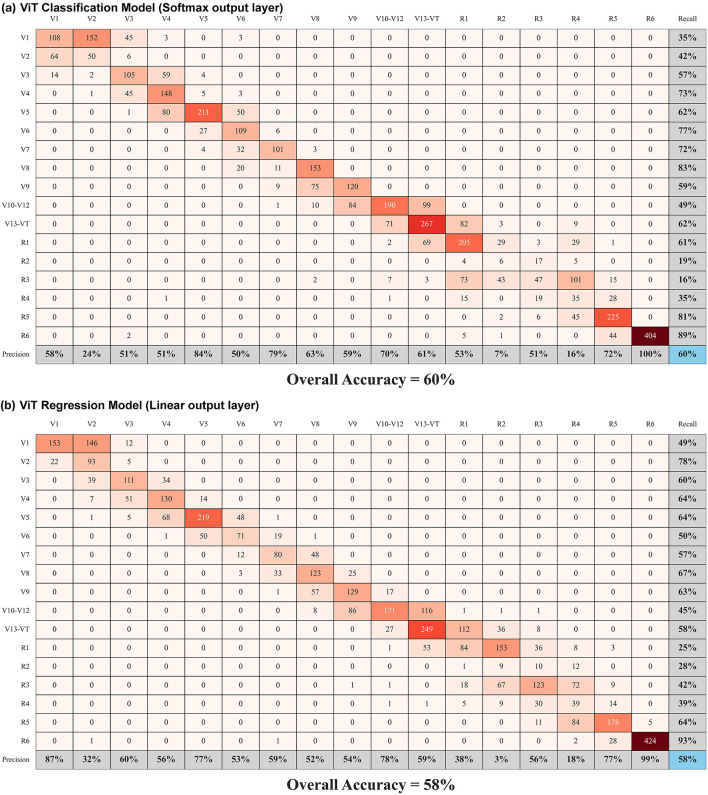
Confusion matrices representing ViT model results with precision, recall, and overall accuracy values. Growth stages V10–V12 and V13–VT have been grouped into a single class.

The results in Table 2 show that the classification model always performs better in terms of classification accuracy while the regression model always performs better with respect to MSE. They also show that using consolidated growth stage labels from V10–V12 and V13–VT improve both classification accuracy and MSE. Figure 12 shows the confusion matrix for both the classification and regression models with the consolidated growth stage labels enumerated. These results are a reminder that ML models optimize to minimize their loss function and that they often perform better with fewer classes that can enable more data samples per class.

**Table 2:** Comparison of ViT model performance metrics.

**Metric**	**ViT classification model**	**ViT regression model**	**ViT classification model consolidated growth stage labels**	**ViT regression model consolidated growth stage labels**
Classification accuracy	52%	45%	60%	58%
Mean squared error	1.815	1.801	0.918	0.705

With that in mind, it is important to consider from a downstream point of view how the results will be used. For the yield estimation use case presented in this paper, using consolidated growth stage labels is expected to provide an appropriate level of fidelity. Likewise, regression is anticipated to be a more appropriate model architecture since it has a lower MSE and also allows for continuous values to be provided to the LSTM model in Figure 6 instead of the discrete integers that would be required by the classification model.

While the agricultural observations in this section would be well understood to agronomists and the ML observations would be well understood by ML engineers, they highlight that a synthesis of both agricultural domain expertise and ML technical expertise is important to develop ML models that are useful in agriculture. While we think there is a human component to encouraging greater interdisciplinary collaboration, we also envision that CI can have some of these agricultural and ML principles built-in to provide a valuable role in guiding the development of useful ML models in agriculture.

### 4.4 Data visualization

In the past decade, we have seen ML make an impact on a myriad of data-rich application areas. As this trend continues, there is a growing need for tools that help practitioners gain a better understanding and trust of the data and insights presented by these technologies (Beauxis-Aussalet et al., 2021). Cultivating trust is critical for success in data-driven and sustainable agriculture (Raturi et al., 2022). Farmers, especially, need validation that data-driven ML tools will be able to achieve their envisioned goals of environmental and economic sustainability (Gardezi et al., 2024). One such tool to improve trust and provide validation is the creation of interactive data visualizations (Beauxis-Aussalet et al., 2021).

**Figure 13:**
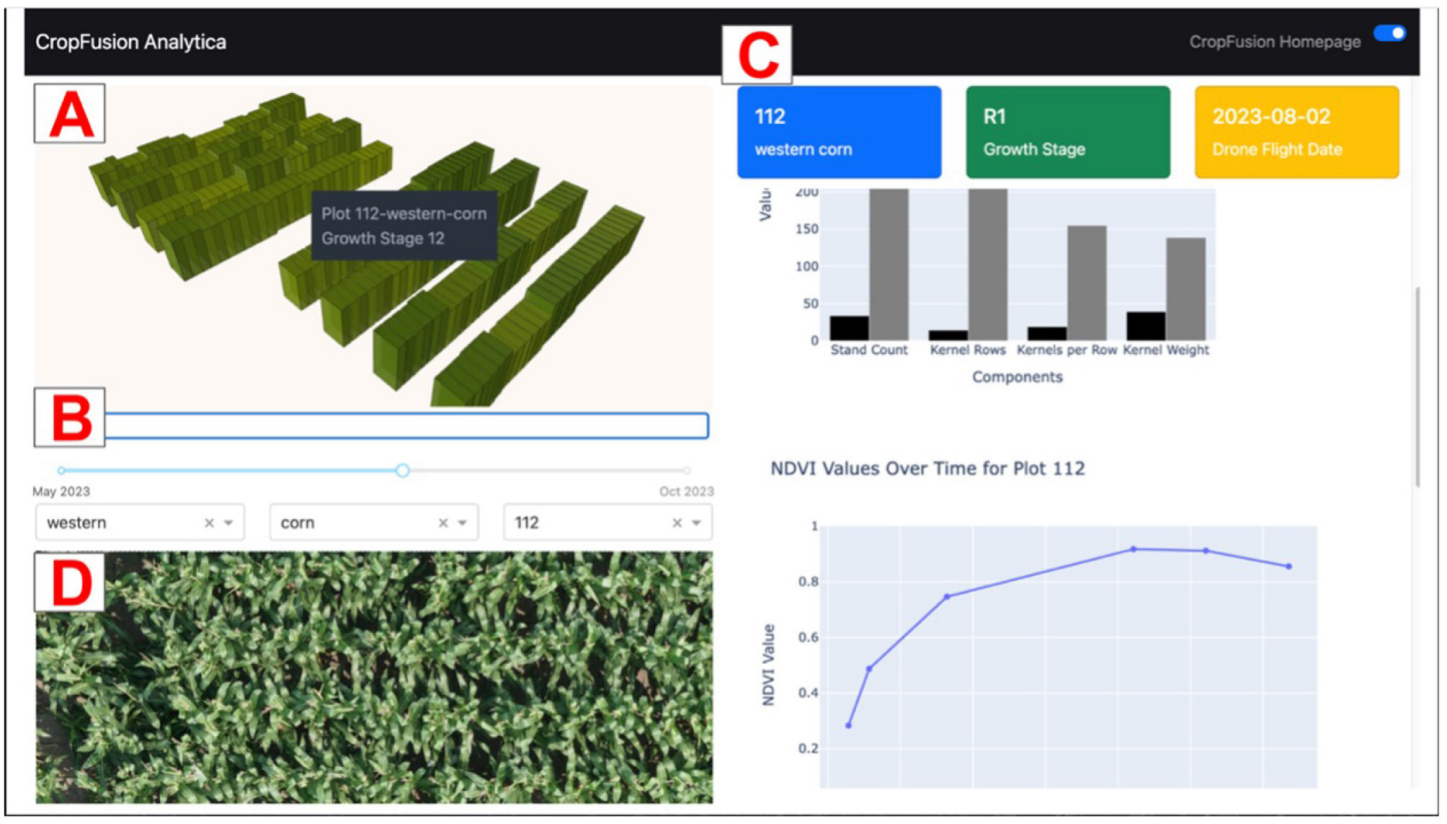
Dashboard Screenshot showing **(A)** Map of a small-plot research field; **(B)** Time slider; **(C)** Dashboard figures; and **(D)** UAS image of selected plot.

As an illustration, we created an interactive data visualization dashboard using the cleaned, wrangled data from this study. Visualization methods at this stage in the ML pipeline are generally used to explore interesting subgroups and pinpoint particular outliers. The purpose of this dashboard is to visualize the collected data at the plot level, providing specific insights for each plot and comparing them to other plots in the field. This provides the user with a reference to see if a particular plot has characteristics that are significantly different from the norm (i.e., out of distribution), enabling a better understanding of the data. Figure 13 shows a screenshot of the dashboard.

The dashboard was created with Plotly Dash, an open source Python framework that enables the creation of interactive, data-driven dashboards. The top left of the dashboard contains a pane with a map focused on a specific field [A]. A 3D geospatial layer is rendered on top of the field using the plot boundaries from the created orthomosaic files (.geojson files). This rendering is created with DeckGL, a WebGL-powered visualization framework. Each plot was outlined and has its own 3D layer. The height and color represent the current crop growth stage of each plot. To view the exact growth stage value of a specific plot, the user may hover over the specific plot.

Below the map, there is a time slider and a series of dropdown menus to select field, crop type, and plot number, respectively [B]. This collection of inputs can be used to update the dashboard figures [C] on the right side of the screen that provides information based on the different modalities of tabular data collected in the study. The upper chart shown in [C] is a chart of yield components (stand count, kernel rows, kernels per row, kernel weight). Each of these combine to account for the overall yield of corn. The lower chart in [C] is a chart of Normalized Difference Vegetation Index (NDVI) values over time for the specific plot. NDVI is a commonly used vegetative index that is calculated from the near infrared (NIR) and red spectral reflectance bands. It is a measure of the health of the vegetation and is calculated by the following formula:


$$\text{NDVI}=\frac{\text{NIR}-\text{RED}}{\text{NIR}+\text{RED}}$$

The position of the time slider can be altered to update the geospatial visualization and figures across different time periods across the growing season. A UAS image of the selected plot at the specific time selected by the time slider is populated at the bottom left of the screen [D].

Perhaps the most significant feature of the dashboard is the interactivity provided in the geospatial visualization. The user may click on a specific plot on the 3D geospatial layer to populate the figures with information about the selected plot, enabling the user to derive plot-specific insights in an interactive, intuitive manner. While the prototype dashboard currently uses plot-scale data for visualization, it can easily be adjusted to incorporate on-farm data. Its interactivity, combined with the time-slider, in the dashboard enables users to explore multimodal data across both spatial and temporal dimensions.

Overall, these figures, driven by plot-clicks and the time-slider, provide a valuable view for users that enables further understanding of the collected data. It can also be utilized to display ML-generated findings alongside ground-truth data, helping to build trust in the ML based models.

## 5 Discussion

In the manuscript, we have introduced some useful methods and approaches relating to AgCI. However, they are still at an early level of maturity and require additional research and development. Furthermore, there is a need to integrate these components into a cohesive CI system for enabling data driven decision making in agriculture. For instance, direct georeferencing techniques show promise to retain original UAS image quality while reducing compute requirements. Further work will be aimed at implementation on high performance computing (HPC) to reduce processing time. Additional work is also needed for error detection to ensure plot tile images are correctly generated. The user interface shows promise, but more user research is needed to tailor the interface to the unique needs of stakeholders. The prototype was built using the prior year's data. A valuable next step will be to implement continuous data processing to show data from the current growing season as data is collected.

Furthermore, there are open questions as to how this work can best be leveraged to serve the needs of agricultural researchers, startups and established companies that serve farmers, and farmers themselves. A primary consideration is lowering the barriers to building datasets of sufficient quality and size. For this reason, collaboration with universities worldwide conducting agricultural research to combine efforts on common needs of AgCI is important.

We hypothesize that various stakeholders will want to engage at varying levels of the technology stack. While some institutions may have the motivation, aptitude, and resources to deploy their own instance of AgCI connected to an HPC backbone, others may prefer to access already existing AgCI for their research needs.

## 6 Conclusion

In accordance with our land-grant university heritage, we advocate that a vibrant community focused on contributing to and using AgCI embodies the mission of land-grant universities to promote agricultural research for the benefit of society. Other institutions across the world may have similar heritage and traditions that provide similar motivations for advancing the field of agriculture.

In summary, this paper articulates the importance of ML applications in agriculture and highlights a data-centric approach to building AgCI. Along the way, it presents some specific approaches that improve data quality, reduce processing time, increase ML model performance, and promote understanding and trust through data visualization. This is all done in the context of three interrelated valuable use cases in agriculture of soil moisture estimation, growth stage estimation, and yield estimation.

We acknowledge that we have only shared learnings from a narrow slice of agricultural use cases. While we have used very tangible examples to illustrate our vision, our vision is not limited to these examples. We believe there is much more agricultural research happening that could be accelerated and be more impactful with access to AgCI connected to an HPC backbone that provides reusable components across data collection, model architecture development, model training, and inference.

The World Wide Web, created as an open standard more than 30 years ago, became the de facto standard over other open and proprietary networks, fundamentally transforming communication and commerce. We draw this analogy because we believe that advancements in ML are ushering in a similarly transformative era. The advancements in ML show promise to be as impactful in the future as the World Wide Web has been over the last 30 years.

We believe this pivotal moment calls for leadership and approaches that develop practical and innovative solutions by synthesizing agricultural domain expertise with the latest advancements in CI and ML technical expertise. We hope that the work presented here inspires discussion and collaboration among various stakeholders (e.g., researchers, crop consultants, farmers) so that the promise of ML in agriculture can be more fully realized by capitalizing on advancements in the ML community at large.

## Data Availability

The raw data supporting the conclusions of this article will be made available by the authors, without undue reservation.
